# Identification of Reference Genes for Quantitative Gene Expression Studies in a Non-Model Tree Pistachio (*Pistacia vera* L.)

**DOI:** 10.1371/journal.pone.0157467

**Published:** 2016-06-16

**Authors:** Maryam Moazzam Jazi, Effat Ghadirzadeh Khorzoghi, Christopher Botanga, Seyed Mahdi Seyedi

**Affiliations:** 1 Plant Biotechnology Department, National Institute of Genetic Engineering and Biotechnology, Tehran, Iran; 2 Department of Biological Sciences, Chicago State University, Chicago, Illinois, United States of America; Flinders University, AUSTRALIA

## Abstract

The tree species, *Pistacia vera* (*P*. *vera*) is an important commercial product that is salt-tolerant and long-lived, with a possible lifespan of over one thousand years. Gene expression analysis is an efficient method to explore the possible regulatory mechanisms underlying these characteristics. Therefore, having the most suitable set of reference genes is required for transcript level normalization under different conditions in *P*. *vera*. In the present study, we selected eight widely used reference genes, *ACT*, *EF1α*, *α-TUB*, *β-TUB*, *GAPDH*, *CYP2*, *UBQ10*, and *18S rRNA*. Using qRT-PCR their expression was assessed in 54 different samples of three cultivars of *P*. *vera*. The samples were collected from different organs under various abiotic treatments (cold, drought, and salt) across three time points. Several statistical programs (geNorm, NormFinder, and BestKeeper) were applied to estimate the expression stability of candidate reference genes. Results obtained from the statistical analysis were then exposed to Rank aggregation package to generate a consensus gene rank. Based on our results, *EF1α* was found to be the superior reference gene in all samples under all abiotic treatments. In addition to *EF1α*, *ACT* and *β-TUB* were the second best reference genes for gene expression analysis in leaf and root. We recommended *β-TUB* as the second most stable gene for samples under the cold and drought treatments, while *ACT* holds the same position in samples analyzed under salt treatment. This report will benefit future research on the expression profiling of *P*. *vera* and other members of the Anacardiaceae family.

## Introduction

Pistachio (*P*. *vera*) is a member of the Anacardiaceae family and is one of the most important commercial products worldwide [1]. According to the 2012 statistics released by the World Food and Agriculture Organization (FAO), Iran is ranked first in pistachio production amongst all major producer countries [2]. In addition to its significant economic value, *P*. *vera* is a salt-tolerant species, making it a suitable alternative crop for cultivation in salinized orchard soils. However, molecular studies have paid little attention to the pistachio tree resulting in no reference genome and only a few EST sequences being in the public data resources.

Gene expression analysis plays a major role in understanding of the complex regulatory networks and identification of genes relevant to new biological processes [3]. Among several techniques available for transcript level measurement, real time quantitative reverse transcription PCR (qRT-PCR) is widely used due to its specificity, sensitivity and dynamic range [4]. Validation of high-throughput technologies such as RNA-sequencing (RNA-seq) through qRT-PCR further implies the robustness of this technique [5]. However, several factors including the quantity and integrity of the extracted RNA, the efficiencies of cDNA synthesis and the products of polymerase chain reaction (PCR) can significantly generate non-specific variations, which should be corrected by using proper controls [6]. Applying the internal control gene known as reference gene or housekeeping gene is the most common way to normalize the transcript level and reduce the inherent experimental errors [6–8]. Therefore, the normalization is a crucial step before performing any qRT-PCR analysis. Ideally, an appropriate reference gene is expected to be stable in terms of expression level across various conditions such as developmental stages, organ types, and experimental conditions [9]. The most frequently used reference genes are the 18S ribosomal RNA (*18SrRNA*), glyceraldehyde-3-phosphate dehydrogenase (*GAPDH*), tubulin (*TUB*), actin (*ACT*), ubiquitin (*UBQ*) and elongation factor (*EF*) [10–12]. They are all expressed at a constant level encoding proteins involved in basic and fundamental cellular processes such as synthesis of ribosome subunits, glycolytic pathway, cytoskeleton components, protein degradation and translation step of mRNAs, respectively [3,13,14]. However, the expression of the reference gene can alter in different organs and experimental conditions, proposing that they may contribute to other cellular processes in addition to primary cell metabolism functions [5]. Hence, there is no universal reference gene for all experiments, and selecting a stably expressed reference gene should be considered for an accurate mRNA level quantification [15–17].

The importance of evaluation of candidate reference genes and subsequent selection of the best genes resulted in the development of several mathematical programs including, geNorm [10], NormFinder [18], and BestKeeper [19] that determine the suitable reference gene(s) by calculating their expression stability. To date, many stable reference genes have been recognized in both model and non-model plant species, such as *Arabidopsis thaliana* [20], *Hordeum vulgare* [21], *Quercus suber* [22], *Cucumis melo* [23], and *Pyrus pyrifolia* [24]. However, no appropriate reference gene has been identified for *P*. *vera*, which limits molecular studies on this species at the transcriptome level.

The objective of this study was to identify the most stably expressed gene(s) across eight commonly used reference genes (*ACT*, *α-TUB*, *β-TUB*, *18S rRNA*, *CYP2*, *EF1α*, *GAPDH*, and *UBQ10*) in roots and leaves of three main pistachio rootstocks, *P*. *vera* L. cultivars Sarakhs, Badami, and Ghazvini under abiotic treatments. Amongst the abiotic stresses, drought, cold, and salinity are the most common limiting factors for plant growth and productivity worldwide. These stresses lead to an average of 50% yield reductions per year [25].

Although the pistachio is able to tolerate the salinity, its yield is severely decreased at the electrical conductivity (EC) value higher than 8 dS/m [26]. Due to the similar signal transduction pathways and plant response mechanisms amongst salinity, cold, and drought stress, these experimental conditions were used as the abiotic treatments to assess the effect of environmental signals on expression variability of the candidate reference genes. In order to estimate the expression stability of candidate reference genes, qRT-PCR was carried out followed by using multiple statistical analyses. The rank aggregation method was also applied to obtain the consensus rank of reference genes. The results demonstrated that none of the selected reference genes is constantly expressed amongst all of the analysed samples. Based on our findings, *EF1α* can be considered as the appropriate reference genes to normalize gene expression analysis of *P*. *vera*. This report will benefit future research on the expression profiling of *P*. *vera* and other members of the Anacardiaceae family.

## Materials and Methods

### Plant materials and stress treatments

The seeds of three pistachio cultivars, including *P*. *vera* L. cultivars Sarakhs, Badami, and Ghazvini as the main indigenous rootstocks of Iran [27], were selected and cultivated at Pistachio Research Institute, located in Rafsanjan, Kerman, Iran. Surface-sterilized seeds were grown in box containing DKW nutrient solution (pH = 5.8) for 16-hour light/8-hour dark photoperiod for 4 weeks. Salt and drought treatments were applied to 4-week-old plants by adding NaCl (300 mM) and polyethylene glycol (PEG) 8000 (27%, w v^-1^) to hydroponic culture medium, respectively. For cold treatment, the plants were subjected to low temperature (4°C) for 6 days.

**Fig 1:**
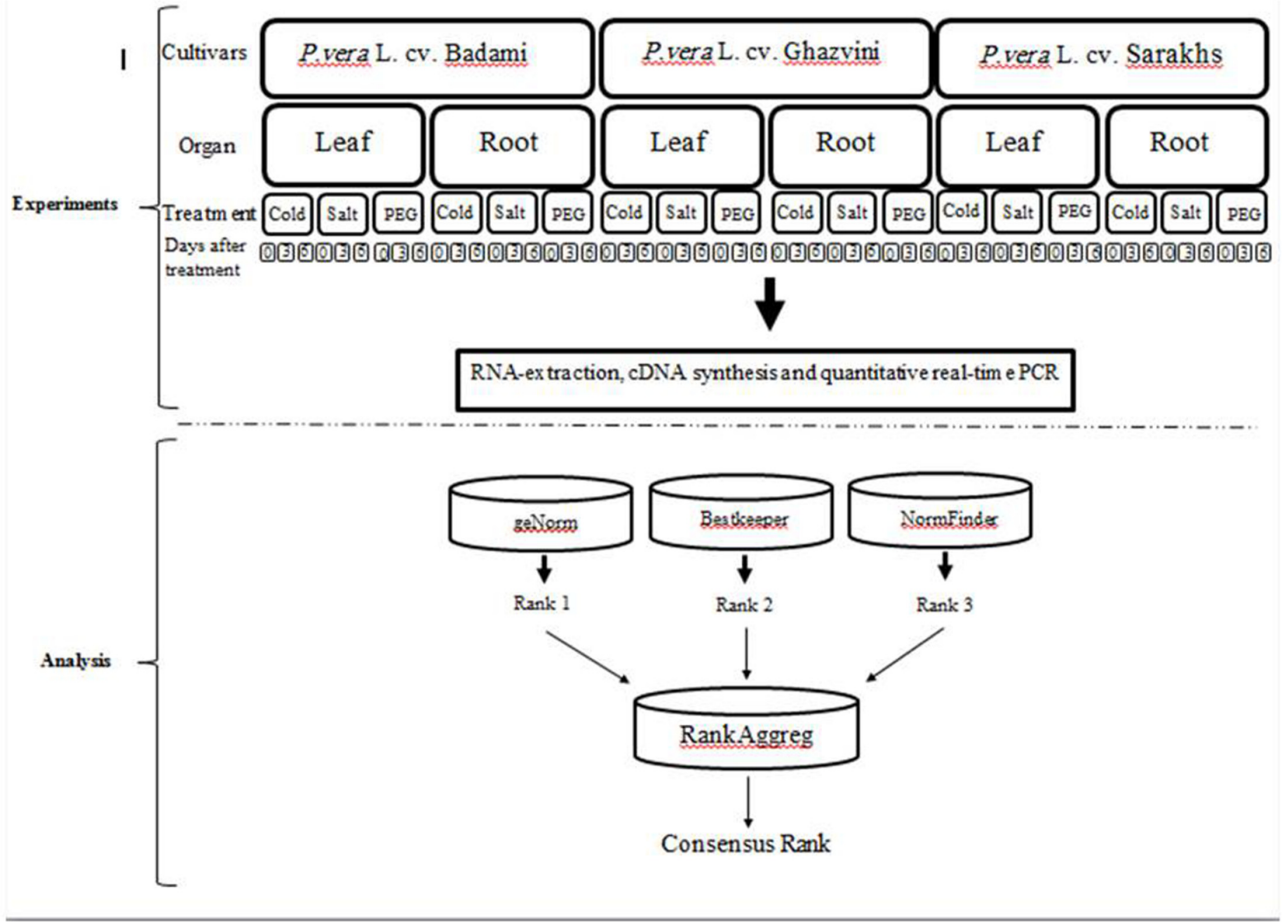
Pipeline for determination of suitable reference gene under different conditions in pistachio. Two organ types (leaf and root) of three pistachio cultivars (*P*. *vera* L. cultivars Sarakhs, Badami, and Ghazvini) under three abiotic treatments (salt, drought, cold) across three time points (0, 3, 6 days after treatment) in three biological replicates were used in this study. Following the RNA extraction, cDNA synthesis and quantitative real-time PCR were conducted. Several statistical programs were applied to rank the reference genes. Different obtained reference gene ranks were subjected in RankAggreg package for attaining a consensus gene rank.

Roots and leaves of plants were collected after 0, 3 and 6 days post treatment. Each experiment was done in triplicate and each replicate had 12 seedlings. In total, 162 diverse samples were collected from the two organ types (leaf and root) of three pistachio cultivars (*P*. *vera* L. cultivars Sarakhs, Badami, and Ghazvini) under three abiotic treatments (salt, drought, cold) across three time points (0, 3, 6 days after treatment) in three biological replicates. All collected samples were immediately frozen in liquid nitrogen and stored at -80°C until use. The workflow of the study is presented in Fig 1.

### RNA extraction and cDNA synthesis

Total RNA was extracted using a modified cetyltrimethylammonium bromide (CTAB) method [1]. Both the quality and quantity of total RNA were determined using spectroscopic measurements at 230, 260, and 280 nm. RNA samples with an absorbance ratio of 1.9–2.2 at OD260/280 and greater than 2 at OD260/230 were used for further analyses. RNA integrity was verified using 2% agarose/formaldehyde gel electrophoresis and ethidium bromide staining.

Single-stranded cDNA was synthesized from 2 μg total RNA using reverse transcriptase and oligo dT primer, according to the manufacturer’s instructions (RevertAid First Strand cDNA Synthesis Kit, Fermentase).

### Cloning the partial sequences of the candidate reference genes

Commonly used reference genes, *ACT*, *α-TUB*, *β-TUB*, *18S rRNA*, *CYP2*, *EF1α*, *GAPDH*, and *UBQ10* were chosen for this study (Table 1). Except *ACT* and *β-TUB* witch their ESTs sequences have been previously reported [1], neither of the sequences of interest has been reported in pistachio. Therefore, the nucleotide sequences of the selected reference genes were obtained from green plants at the Gene Bank of the National Center for Biotechnology Information (NCBI). Across the sequences, the conserved segments were characterized through the sequence alignments by MegAlign software and used for designing the PCR primers via Oligo7 program.

**Table 1:** List of the selected widely used reference genes.

Gene symbol	Gene name	Function
*18SrRNA*	18S ribosomal RNA	Cytosolic small ribosomal subunit, translation
*ACT*	Actin	Cytoskeletal structural protein
*GAPDH*	Glyceraldehyde-3-phosphate dehydrogenase	Oxidoreductase in glycolysis and gluconeogenesis
*β-TUB*	Tubulin beta	Structural constituent of cytoskeleton
*α-TUB*	Tubulin alpha	Structural constituent of cytoskeleton
*UBQ10*	Ubiquitin 10	Protein-binding protein modification process
*EF1α*	Elongation factor 1-*α*	Translation elongation factor activity
*CYP2*	Cyclophilin 2	Peptidyl-prolyl cis-trans isomerase activity

The conditions of PCR reaction conditions were set as: the initial denaturation at 94°C for 3 minutes (min), followed by 30 cycles of denaturation at 94°C for 30 seconds (s), annealing at 55°C-58°C for 30 s and extension at 72°C for 50 s with a final extension at 72°C for 10 min. The amplified products were resolved on 1.2% agarose gel. Amplicons were purified using Gel Extraction Kit (Roch, Germany) and ligated into the pTG19-T vector according to the manufacturer’s instructions (InsTAclone PCR Cloning Kit, Fermentas). The constructed plasmids were subsequently introduced into *Escherichia coli* DH5α competent cells and spread on X-gal/IPTG plate. White colonies were picked after 15 hours of incubation at 37°C and the corresponding DNA was amplified through colony PCR to confirm positive colonies. Finally, the colonies of interest were grown in Luria Bertani (LB) liquid culture. The plasmids were isolated using the Plasmid Extraction Kit (Roch, Germany) and sequenced. The resulting sequences were compared to the available sequences in NCBI using the BLASTn algorithm. The verified sequences were submitted to NCBI and corresponding accession numbers were acquired.

### QRT-PCR primer design and testing

Based on the obtained partial gene sequences, eight gene-specific primer sets were designed (Table 2) using Oligo7 software. The following parameters were used for qRT-PCR: amplicon length from 100 to 200 bp and a melting temperature (Tm) of 60°C-63°C, incapable of forming stable secondary structures as well as mispriming. Amplification efficiency (E) was calculated based on the slope of the standard curve using the following equation: (E = 10 ^-1/slope - 1^). All reactions were performed using a ten-fold dilution series over at least four dilution points that were measured in triplicate. Primer specificity was verified by analysis of the melting-curve following qRT-PCR and the products were evaluated using agarose gel electrophoresis.

**Table 2:** Primer sequences for quantitative real-time PCR of selected reference genes.

Accession number	Gene symbol	Forward primer sequence 5´-3´	Reverse primer sequence 5´-3´	Tm (°C)
KT188931	*18SrRNA*	GGTCTGTGATGCCCTTAGATG	GATCTATCCCCATCACGATGA	60
JZ896671	*ACT*	GTATCCACGAGACCACCTACA	GGAGCAACGACCTTGATCTTC	60
JZ896666	*α-TUB*	GCTGATAACTGCACTGGTCTTC	CCTGAGGAGATGGGTAAACAG	60
JZ896668	*GAPDH*	CAGCAATGCTTCTTGCACCACA	CGGTGTAGGAGTGAGTAGTT	60
JZ896672	*β-TUB*	TGGGACCCACGTGAAGTCAG	GAGTGGTGTAACTTGCTGCTTG	60
JZ896669	*UBQ10*	AGAAGGAGTCAACCCTCCATC	CTCTCGACCTCAAGAGTGATG	60
JZ896667	*EF1α*	GGCAAGGTATGATGAAATCGTG	ATCACCCTCAAATCCAGAGATG	63
JZ896670	*CYP2*	ATGGAACGGGAGGTGAATCTG	GAGCCACTCCGTCTTAGCTG	60

### Quantitative real-time PCR

All cDNA samples were diluted 10-fold with RNase-free water and real-time PCR reactions were performed using a Rotor-Gene 6000 thermocycler (Qigen, USA) and the SYBR Green real-time PCR master mix (Ampliqon, Denmark). The reaction mixture contained 1.0 μL of diluted cDNA sample, 0.5 μL of each of the forward and reverse primers (10 μM) and 10 μL real-time master mix with a final volume of 20 μL.

Each PCR reaction had a reverse transcription negative control and a negative control with no template in order to check the potential genomic DNA and reagents contamination, respectively. QRT-PCR conditions were conducted as follow: initial denaturation and polymerase activation step at 94°C for 15 min, followed by 40 cycles 94°C for 30 s, 63°C for 30 s and 72°C for 20 s. A melting-curve analysis was: 65°C to 95°C, with fluorescence measured every 0.5°C at the end of each reaction to further confirm the specificity of the primer pairs.

### Statistical analysis of gene expression stability

Three popular software, geNorm [10], NormFinder [18] and BestKeeper [19] were used to determine the most stable reference genes under the experimental conditions. In general, the principle of all the programs is determination of the expression stability index. Furthermore, the RankAggreg, an R package was applied to combine the stability results derived from the three applied tools and to identify a consensus rank for the reference genes [28].

## Results

Data normalization using a set of reference genes is a crucial procedure during the evaluation of the expression level of target genes in various tissues or under different conditions using qRT-PCR. In the present study, a SYBR green-based qRT-PCR assay was carried out on eight candidate reference genes (*GAPDH*, *UBQ10*, *ACT*, *CYP2*, *α-TUB*, *β-TUB*, *EF1α* and *18S rRNA*) using 162 diverse samples of *P*. *vera* in order to identify the most stable reference gene(s). Due to the lack of pistachio sequence for the genes of interest, gene amplification was performed using primers designed from the conserved segments of the gene sequences of the other plants. The PCR product was sequenced to confirm the desired sequences and to attain the partial nucleotide sequences of each candidate gene in pistachio. The gene specific primers were designed to accurately amplify the candidate reference gene using qRT-PCR. All the primer pairs amplified a single PCR product of the expected size, which appeared as one peak on the melting curve.

We applied different organs, treatments and cultivars to monitor their influences on the reference gene expression level and to select the most stably expressed gene under a wide range of conditions. In order to perform the comprehensive expression analysis of candidate reference genes, the samples were divided into 6 subsets according to the organ type and treatment conditions. The subsets were consisted of control, cold, salt, and drought treatments (roots and leaves of three cultivars for each condition), root subset (roots from control and abiotic treatments of three cultivars), and leaf subset (leaves from control and abiotic treatments of three cultivars). Additionally, organ and treatment subsets were considered together as the whole dataset (Table 3).

**Table 3:** Summary of the different conditions comprising the cultivar, organ, and abiotic treatment, time course and whole dataset were used in present study. Each experiment was performed in triplicate.

Cultivar	Organ	Treatment	Time	Leaf	Root	Control	Cold	Drought	Salt	Whole
Badami	Leaf	cold	0	+		+				+
			3	+						+
			6	+						+
		Drought	0	+		+				+
			3	+				+		+
			6	+				+		+
		Salt	0	+		+				+
			3	+					+	+
			6	+					+	+
	Root	Cold	0		+	+				+
			3		+		+			+
			6		+		+			+
		Drought	0		+	+				+
			3		+			+		+
			6		+			+		+
		Salt	0		+	+				+
			3		+				+	+
			6		+				+	+
Ghazvini	Leaf	Cold	0	+		+				+
			3	+			+			+
			6	+			+			+
		Drought	0	+		+				+
			3	+				+		+
			6	+				+		+
		Salt	0	+		+				+
			3	+					+	+
			6	+					+	+
	Root	Cold	0		+	+				+
			3		+		+			+
			6		+		+			+
		Drought	0		+	+				+
			3		+			+		+
			6		+			+		+
		Salt	0		+	+				+
			3		+				+	+
			6		+				+	+
Sarakhs	Leaf	cold	0	+		+				+
			3	+			+			+
			6	+			+			+
		Drought	0	+		+				+
			3	+				+		+
			6	+				+		+
		Salt	0	+		+				+
			3	+					+	+
			6	+					+	+
	Root	Cold	0		+	+				+
			3		+		+			+
			6		+		+			+
		Drought	0		+	+				+
			3		+			+		+
			6		+			+		+
		Salt	0		+	+				+
			3		+				+	+
			6		+				+	+

**Fig 2:**
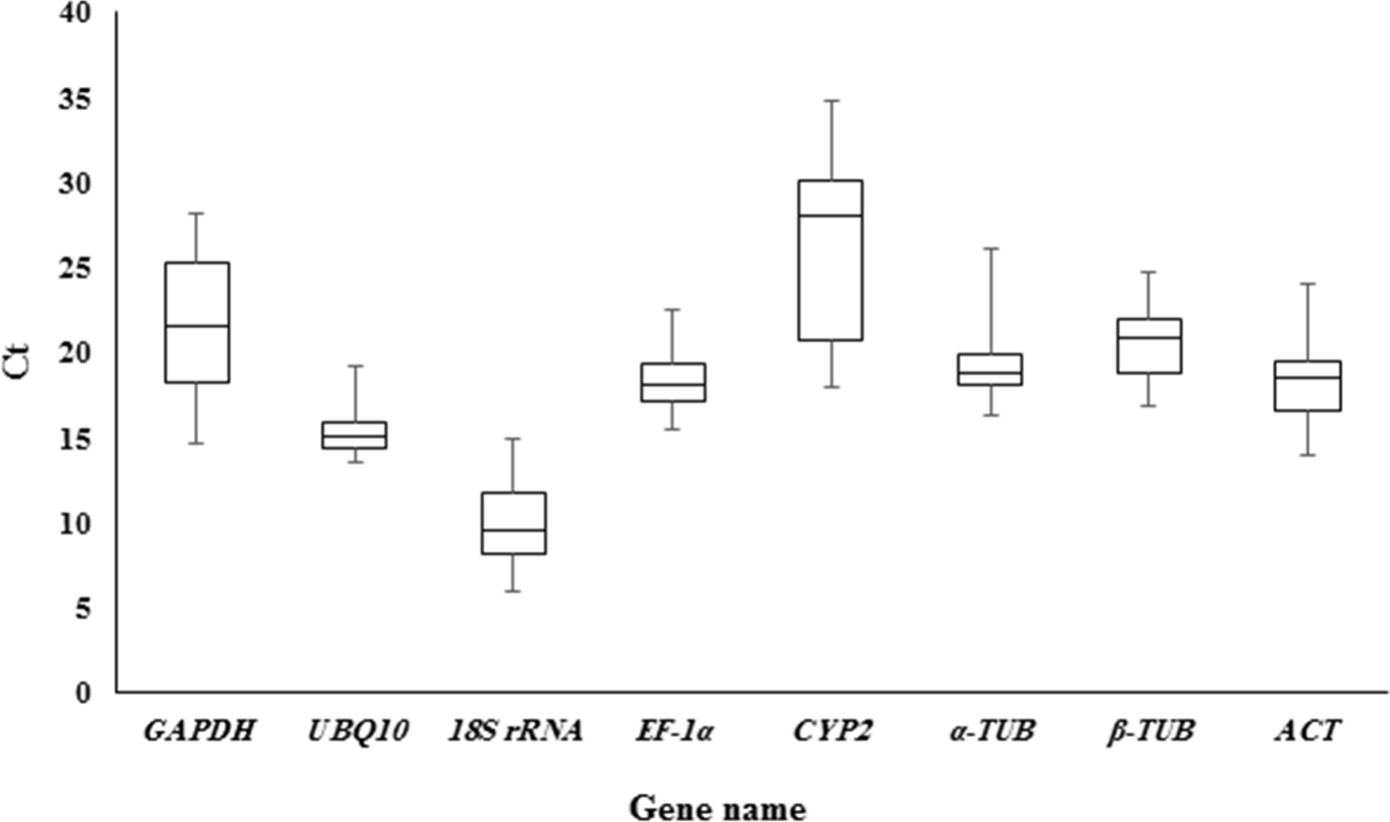
The distribution of gene expression levels of eight candidate reference genes in whole dataset. The boxes represent mean Ct values and bars correspond to the standard deviation.

**Fig 3:**
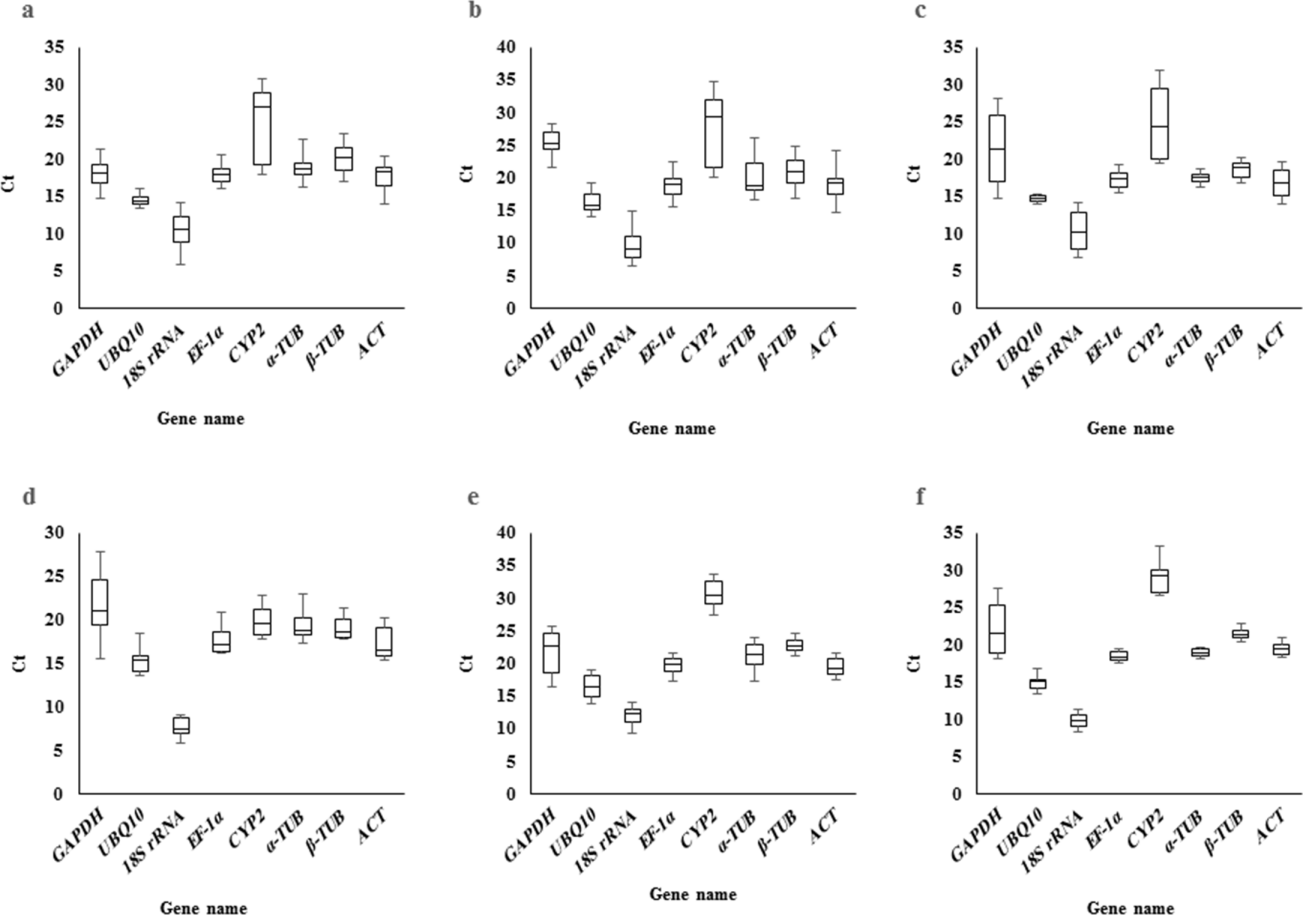
The distribution of gene expression levels of eight candidate reference genes in different subsets. The boxes represent mean Ct values and bars correspond to the standard deviation from various subset including: leaf subset (a), root subset (b), control subset (c), cold subset (d), drought subset (e), and salt subset (f).

The cycle threshold (Ct) value that represents the cycle, at which a significant increase of the PCR product occurs, was calculated to determine the gene expression variability [8,29]. The Ct value of candidate reference genes with their standard error (SE) is presented in Table 4. The Ct values reflect the large gene expression variation across the whole dataset, containing all abiotic treatments and organs (Fig 2). *CYP2* exhibited the highest expression variation with the average Ct value of 26.16, whereas *UBQ10* had the lowest expression variation and mean Ct value of 15.42 (Fig 2). Similarly, box-and-whiskers plots were traced for each subset to determine the expression variability of selected reference genes (Fig 3). As illustrated in Fig 3, the average Ct values of most of the candidate reference genes ranged from 6 to 28 cycles and none of them were constantly expressed across the tested subsets. Hence, recognition of an appropriate reference gene through statistical analysis is required for accurate transcriptome analysis in pistachio.

**Table 4:** Mean of the cycle threshold (Ct) and their standard error (SE) for the eight reference genes in different organs (A and B) under various conditions (C, D, E and F) as well as whole dataset (G).

Reference gene	Leaf (A)	Root (B)	Control (C)	Cold (D)	Drought (E)	Salt (F)	Whole dataset (G)
*18S rRNA*	10.45±0.31	9.64±0.34	10.41±0.51	7.70±0.21	12.23±0.31	9.83±0.18	10.04±0.23
*ACT*	17.83±0.24	18.79±0.32	16.81±0.38	17.32±0.34	19.70±0.37	19.41±0.17	18.31±0.21
*α-TUB*	18.84±0.19	19.93±0.37	17.52±0.13	19.51±0.36	21.55±0.49	18.96±0.1	19.38±0.21
*GAPDH*	18.08±0.26	25.49±0.24	21.45±1.08	21.67±0.82	21.83±0.73	22.19±0.71	21.78±0.42
*β-TUB*	20.12±0.26	20.81±0.32	18.54±0.22	19.09±0.26	22.81±0.21	21.44±0.14	20.47±0.21
*UBQ10*	14.50±0.09	16.34±0.21	14.74±0.08	15.46±0.31	16.53±0.37	14.97±0.21	15.42±0.15
*EF1α*	17.87±0.18	18.70±0.26	17.20±0.24	17.96±0.33	19.76±0.31	18.47±0.14	18.28±0.16
*CYP2*	24.86±0.7	27.46±0.74	24.93±1.02	19.91±0.35	30.76±0.46	29.04±0.38	26.16±0.53

Each reaction was conducted in triplicate and the mean of corresponding Ct values were calculated.

The emergence of fluorescence signal after 38.5 cycles in negative control with no template in all tested samples implied that PCR reagents are free of any contamination. Furthermore, the fluorescence signal appeared after 39.7 cycles in the reverse transcription negative control suggesting extracted total RNA are free of genomic DNA contamination.

The expression stability of the eight candidate reference genes in each subset was measured and ranked using three different statistical algorithms namely geNorm (NormqPCR), NormFinder, and Bestkeeper. RankAggreg package was also applied in order to merge the varied ranked gene lists resulted from the mentioned three programs. geNorm analysis has been employed by NormqPCR, an R-based package. geNorm algorithm considers all samples as a single population and calculates a measure of expression stability value (M) based on the average pairwise variation between all tested reference genes. In the algorithm, stepwise exclusion of the least stable gene allowed the genes to be ranked according to their M value (the M-value cut-off of 1.5) [30]. According to our results obtained from geNorm, M values of high-ranked genes were less than the geNorm threshold of 1.5 (Table 5), indicating that these candidate genes had stable expression levels. As presented in Table 5, when the whole dataset was considered, *ACT* and *EF1α* were the most stably expressed reference genes with M-value of 0.9. In comparison, the M-value of *CYP2*, *GAPDH*, *18S rRNA* were above the cutoff value, representing their least expression stability.

**Table 5:** Candidate reference genes rank in term of their expression stability as computed by geNorm.

Rank position	Leaf (A)	Root (B)	Control (C)	Cold (D)	Drought (E)	Salt (F)	Whole dataset
1	*ACT*	*ACT*	*β-TUB*	*β-TUB*	*β-TUB*	*ACT*	*ACT*
M-value	0.79	1	0.56	0.44	0.63	0.37	0.9
2	*EF1α*	*EF1α*	*EF1α*	*EF1α*	*EF1α*	*EF1α*	*EF1α*
M-value	0.79	1	0.56	0.44	0.63	0.37	0.9
3	*α-TUB*	*β-TUB*	*α-TUB*	*ACT*	*UBQ10*	*UBQ10*	*β-TUB*
M-value	1	1.12	0.76	0.51	0.76	0.62	1.07
4	*GAPDH*	*UBQ10*	*ACT*	*α-TUB*	*ACT*	*α-TUB*	*α-TUB*
M-value	1.09	1.26	0.97	0.61	0.87	0.75	1.21
5	*β-TUB*	*α-TUB*	*UBQ10*	*UBQ10*	*α-TUB*	*β-TUB*	*UBQ10*
M-value	1.16	1.35	1.15	0.75	1.04	0.95	1.32
6	*UBQ10*	*GAPDH*	*18S rRNA*	*CYP2*	*18S rRNA*	*18S rRNA*	*18S rRNA*
M-value	1.27	1.65	1.36	0.92	1.33	1.05	1.63
7	*18S rRNA*	*18S rRNA*	*CYP2*	*18S rRNA*	*CYP2*	*CYP2*	*GAPDH*
M-value	1.46	1.86	2.03	1.26	1.6	1.25	2.21
8	*CYP2*	*CYP2*	*GAPDH*	*GAPDH*	*GAPDH*	*GAPDH*	*CYP2*
M-value	2.05	2.42	2.75	1.67	1.82	1.72	2.72

Expression stability and ranking of eight reference genes as calculated by geNorm in different organs (A and B) under various conditions (C, D, E and F) as well as whole dataset (G). Reference genes with M values of less than the geNorm threshold of 1.5 were considered as the most stably expressed genes.

“NormFinder” is an ANOVA-based algorithm that computes a stability value for the reference genes. It calculates intra-group variability for the genes within each group, the inter-group variability between the groups and combines both of these results in a stability value for each tested gene [47]. The most stable gene expression is specified by the lowest expression stability value [30]. The stability values of whole dataset created by Normfinder analysis are listed in Table 6. Consistent with the geNorm output, *EF1α* and *ACT* were ranked as the most stable reference gene with stability value of 0.02, while *18S rRNA* had the highest stability value of 0.2 (Table 6). Therefore, *18S rRNA* was the least stable gene across all candidate reference genes.

**Table 6:** Candidate reference genes rank in term of their expression stability as computed by NormFinder.

Rank position	Leaf (A)	Root (B)	Control (C)	Cold (D)	Drought (E)	Salt (F)	Whole dataset (G)
1	*ACT*	*EF1α*	*β-TUB*	*UBQ10*	*EF1α*	*EF1α*	*EF1α*
Stability value	0.015	0.026	0.031	0.007	0.019	0.009	0.026
2	*β-TUB*	*β-TUB*	*ACT*	*β-TUB*	*β-TUB*	*ACT*	*ACT*
Stability value	0.03	0.03	0.032	0.009	0.03	0.012	0.028
3	*EF1α*	*ACT*	*EF1α*	*α-TUB*	*ACT*	*α-TUB*	*β-TUB*
Stability value	0.03	0.044	0.038	0.018	0.03	0.029	0.033
4	*α-TUB*	*UBQ10*	*α-TUB*	*EF1α*	*UBQ10*	*UBQ10*	*α-TUB*
Stability value	0.067	0.082	0.072	0.027	0.03	0.033	0.073
5	*GAPDH*	*α-TUB*	*CYP2*	*ACT*	*α-TUB*	*CYP2*	*UBQ10*
Stability value	0.087	0.083	0.121	0.029	0.051	0.037	0.086
6	*UBQ10*	*GAPDH*	*UBQ10*	*CYP2*	*CYP2*	*β-TUB*	*CYP2*
Stability value	0.092	0.106	0.124	0.045	0.08	0.049	0.142
7	*CYP2*	*CYP2*	*18S rRNA*	*GAPDH*	*GAPDH*	*18S rRNA*	*GAPDH*
Stability value	0.152	0.137	0.182	0.148	0.111	0.127	0.180
8	*18S rRNA*	*18S rRNA*	*GAPDH*	*18S rRNA*	*18S rRNA*	*GAPDH*	*18S Rrna*
Stability value	0.172	0.193	0.238	0.197	0.149	0.152	0.205

Expression stability and ranking of eight reference genes as calculated by NormFinder in different organs (A and B) under various conditions (C, D, E and F) as well as whole dataset (G). Reference genes with the lowest expression stability value identified as the most stably expressed genes.

BestKeeper, an Excel-based tool, evaluates the stability of reference gene expression based on the coefficient of correlation to the BestKeeper index, which is the geometric mean of the Ct values of all candidate reference genes [19,31]. It also calculates the coefficient of variance (CV) and the standard deviation (SD) of the Ct values [19]. In this algorithm, reference genes are identified with the highest expression stability by exhibiting the lowest standard deviation (SD). Upon analysis using BestKeeper in the whole dataset, *UBQ10* and *EF1α* were recognized as the most stably expressed genes with SD values of 1.1 and 1.3, respectively. *CYP2* and *GAPDH* with SD values of 4.6 and 3.7 were determined to be the least stably expressed genes (Table 7).

**Table 7:** Candidate reference genes rank in term of their expression stability as computed by BestKeeper.

Rank position	Leaf (A)	Root (B)	Control (C)	Cold (D)	Drought (E)	Salt (F)	Whole dataset (G)
1	*UBQ10*	*UBQ10*	*UBQ10*	*18S rRNA*	*β-TUB*	*α-TUB*	*UBQ10*
SD	0.54	1.27	0.35	0.89	0.84	0.39	1.13
2	*α-TUB*	*GAPDH*	*α-TUB*	*β-TUB*	*EF1α*	*β-TUB*	*EF1α*
SD	0.96	1.38	0.55	1.13	1.19	0.56	1.3
3	*EF1α*	*EF1α*	*β-TUB*	*UBQ10*	*18S rRNA*	*ACT*	*ACT*
SD	0.99	1.55	0.97	1.18	1.22	0.64	1.59
4	*ACT*	*ACT*	*EF1α*	*EF1α*	*ACT*	*EF1α*	*α-TUB*
SD	1.39	1.73	1.05	1.4	1.47	0.65	1.6
5	*GAPDH*	*β-TUB*	*ACT*	*α-TUB*	*UBQ10*	*UBQ10*	*β-TUB*
SD	1.43	1.8	1.77	1.44	1.6	0.78	1.72
6	*β-TUB*	*18S rRNA*	*18S rRNA*	*ACT*	*CYP2*	*18S rRNA*	*18S rRNA*
SD	1.58	1.97	2.32	1.5	1.94	0.81	1.94
7	*18S rRNA*	*α-TUB*	*CYP2*	*CYP2*	*α-TUB*	*CYP2*	*GAPDH*
SD	1.71	2.19	4.77	1.58	1.95	1.49	3.71
8	*CYP2*	*CYP2*	*GAPDH*	*GAPDH*	*GAPDH*	*GAPDH*	*CYP2*
SD	4.55	4.69	4.96	3.29	3.29	3.29	4.62

Expression stability and ranking of eight reference genes as calculated by BestKeeper in different organs (A and B) under various conditions (C, D, E and F) as well as whole dataset (G). Reference genes with the lowest standard deviation (SD) are recognized as the genes with the highest expression stability.

To detect the organ-specific and experimental-condition-specific reference genes, all statistical analyses were conducted for each subset. In leaf subset, *ACT* and *EF1α* showed the most stability using geNorm with the M-value of 0.8; similarly, *ACT* was the best in NormFinder with the stability value of 0.015 (Tables 5 and 6). Contrary to geNorm and NormFinder outputs, BestKeeper returned a varied ranked gene list, in which *UBQ10* with the SD value of 0.5 was found as the most stable gene (Table 7). *CYP2* was characterized as the least stable gene by three types of algorithm (Tables 5, 6 and 7).

In root subset, *ACT* and *EF1α* with the M-value of 1 were both distinguished as the genes with the highest expression stability in geNorm (Table 5). The lowest stability value (0.02) was assigned to *EF1α* by NormFinder, to be specified as the most stable gene (Table 6). BestKeeper analysis indicated that *UBQ10* with SD value of 0.5 had the least variation among all tested reference genes (Table 6). *CYP2* and *18S rRNA* were proved to be the genes with the least expression stability by three softwares (Tables 5, 6 and 7).

We observed that the expression variability of candidate reference genes differed among samples under different abiotic treatments. In control subset, geNorm specified that *EF1α* and *β-TUB* were the most stably expressed reference genes. In addition to *EF1α* and *β-TUB*, *ACT* also exhibited the highest stability in NormFinder (Tables 5 and 6).

There were some discrepancies between the BestKeeper results with those obtained by geNorm and NormFinder. BestKeeper proved *UBQ10* with the lowest SD value of 0.3 to be the best reference gene (Table 7). All used statistical analysis revealed *GAPDH* to be the lowest stable reference gene (Tables 5, 6 and 7).

Using geNorm, the top-ranked reference genes were *β-TUB* and *EF1α* in drought and cold subsets, while the similar situation had been assigned to *EF1α* and *ACT* with the M-value of 0.37 in salt subset (Table 4). The results of NormFinder showed that the most stable gene in the salt and drought subsets was *EF1α* with stability values of 0.009 and 0.02, respectively. In the cold subset, *UBQ10* had the lowest stability value (0.007), which placed this gene on the first rank position (Table 6). According to BestKeeper analysis, *α-TUB*, *β-TUB* and *18S rRNA* acquired the highest rank across all candidate reference genes in the salt, drought, and cold subsets, respectively (Table 7). Interestingly, all algorithms established *GAPDH* as the lowest stably expressed reference gene throughout all abiotic treatment subsets (Tables 5, 6 and 7).

We observed some differences in the ranked gene list achieved from the various software used (Tables 5–7). These inconsistencies could be related to differences in the embedded algorithms in each software. Therefore, we applied RankAggreg, an R-based package to combine the ranking gene lists produced by the each software in order to determine the consensus rank [28]. Based on our rank aggregation results, *EF1α* and *ACT* have been demonstrated as the genes with highest expression stability in the leaf and salt subsets as well as in the whole dataset, whereas *EF1α* and *β-TUB* are the best reference genes in the root, control, and drought subsets. For the cold subset, *UBQ10* and *β-TUB* were found as the most appropriate reference genes; however, *EF1α* was ranked as the third-most stable gene in this subset (Table 8). Overall, *EF1α* was located in the top rank in all subsets using rank aggregation analysis.

**Table 8:** Consensus ranking of candidate reference gene in term of their expression stability as computed by RankAggreg.

Rank position	Leaf (A)	Root (B)	Control (C)	Cold (D)	Drought (E)	Salt (F)	Whole dataset (G)
1	*ACT*	*EF1α*	*β-TUB*	*UBQ10*	*β-TUB*	*EF1α*	*EF1α*
2	*EF1α*	*β-TUB*	*EF1α*	*β-TUB*	*EF1α*	*ACT*	*ACT*
3	*α-TUB*	*ACT*	*α-TUB*	*EF1α*	*UBQ10*	*α-TUB*	*β-TUB*
4	*GAPDH*	*UBQ10*	*ACT*	*α-TUB*	*ACT*	*UBQ10*	*α-TUB*
5	*β-TUB*	*α-TUB*	*UBQ10*	*ACT*	*α-TUB*	*β-TUB*	*UBQ10*
6	*UBQ10*	*GAPDH*	*18S rRNA*	*CYP2*	*18S rRNA*	*18S rRNA*	*18S rRNA*
7	*18S rRNA*	*18S rRNA*	*CYP2*	*18S rRNA*	*CYP2*	*CYP2*	*GAPDH*
8	*CYP2*	*CYP2*	*GAPDH*	*GAPDH*	*GAPDH*	*GAPDH*	*CYP2*

Varied reference gene ranks achieved by three softwares were subjected to RankAggreg package to determine consensus rank.

## Discussion

Gene expression profiling provides new insights into regulatory gene networks and offers the possibility of understanding of various biological processes [3]. QRT-PCR is one of the widely used techniques to assess gene expression variation [3,32,33]. However, accurate gene expression analysis is required to identify the superior reference gene for transcript normalization.

To our knowledge, this is the first report to assess the expression variability of candidate reference genes in *P*. *vera* using qRT-PCR. Our results demonstrated a wide variation of Ct values among the tested reference genes as presented on the distribution plot (Figs 2 and 3). Consequently, the evaluation of reference gene expression variability is of great importance. Several studies have observed that the majority of reference genes are significantly influenced by experimental conditions [11,34–36]. The most plausible explanation is that reference genes are not only involved in basal cell metabolism but also contribute to other cellular functions [37,38]. For example, ubiquitin is involved in non-proteolytic activities besides its initial role in proteolytic degradation. Similarly, actin is not only a main component of the eukaryotic cytoskeleton, but also participates in cell division and the distribution of plasma membrane proteins [31,39]. Currently, the advent of various statistical algorithms like geNorm, BestKeeper, and NormFinder have facilitated the selection of appropriate reference genes for a given experimental condition [31]. Our statistical analysis demonstrated the varied patterns of most stable reference genes across all experimental subsets. This result is in agreement with previous studies on other plants, such as grape [40], bamboo [41], peach [42], and eggplant [43]. This finding could be interpreted from two points of view: first, no single reference gene can be used for every biological experiment to accurately normalize expression data. Second, applying different operative algorithms within each software can result in diverse reference gene ranking [36,44]. In our study, geNorm and NormFinder algorithms reported *EF1α* and *ACT* as the highest stable reference genes when the whole dataset was examined, whereas BestKeeper ranked *UBQ10* as the most stable. In general, outputs of geNorm and NormFinder programs were relatively closer in comparison with the BestKeeper results. This could be due to the fact that geNorm and NormFinder have considered the inter-group and intra-group correction, contrary to BestKeeper, which can make the expected discrepancy [45]. A similar observation has been reported by Jiang et al in 2014 during their study to establish a valid reference gene for *Oenanthe javanica* [46].

With respect to several statistical algorithms used in this study, one might wonder which one could lead to the most reliable results. To resolve this issue, the RankAggreg package could be employed to acquire the consensus ranking of reference genes [44,47]. This package uses the matrix of rank-ordered genes as an input to unify previously calculated expression stability levels [48]. When we executed this analysis on each subset, *EF1α* and *ACT* were located among the four best genes. For data normalization it is recommended that at least two valid reference genes are used [36]. Therefore, we attempted to detect the second suitable refernce gene for the given conditions. In addition to *EF1α* (the most stabley expressed reference gene in all subsets), we identified *ACT* and *β-TUB* as the second best reference genes for gene expression analysis in leaf and root, respectively. Furthermore, *β-TUB* is recommended as the second most stable gene for transcript profiling under cold and drought treatments, while *ACT* holds the same position in samples analyzed under salt treatment. Overall, by holding the first and second positions, *EF1α* and *ACT* were found to be the suprior reference genes in the whole dataset that covers all abiotic treatments and organ subsets. Tong et al (2009) reported that *EF1α* was constantly expressed in all peach samples under different conditions [42]. Ma et al (2013) considered the expression stability of nine reference genes under different treatments in soybean and stated that besides *EF1α*, *ACT* was also among the top ranked-genes in different samples following salinity and drought treatments [49]. These results are consistent with our findings, proposing that *EF1α* and *ACT* could be presumed as the applicable reference genes for accurate normalization purposes in pistachio. In particular, *EF1α* was proved to be stably expressed under different situations in various studies [50]. This is potentially due to *EF1α* having a major role in facilitating the binding of aminoacyl-tRNA to the ribosome during the elongation step [51].

## Conclusion

The current study represents the first attempt to identify the best reference gene for the quantification of transcript levels in pistachio. Based on our findings, *EF1α* was the most stably expressed gene amongst the tested candidate genes when expression variation was estimated using various statistical analyses in all subsets. Interestingly, this gene had the highest ranking based on rank aggregation analysis. However, we found that *GAPDH* and *CYP2* could not be applied for correcting experimental errors during gene expression analysis in *P*. *vera*. Our results provide a guideline to select a valid reference gene for gene expression analyses in future studies.
